# Manipulating Intracellular Oxidative Conditions to Enhance Porphyrin Production in *Escherichia coli*

**DOI:** 10.3390/bioengineering12010083

**Published:** 2025-01-17

**Authors:** Bahareh Arab, Murray Moo-Young, Yilan Liu, C. Perry Chou

**Affiliations:** Department of Chemical Engineering, University of Waterloo, 200 University Avenue West, Waterloo, ON N2L 3G1, Canada; barab@uwaterloo.ca (B.A.); mooyoung@uwaterloo.ca (M.M.-Y.); yilan.liu@uwaterloo.ca (Y.L.)

**Keywords:** antioxidant, *Escherichia coli*, porphyrin, strain engineering, reactive oxygen species

## Abstract

Being essential intermediates for the biosynthesis of heme, chlorophyll, and several other biologically critical compounds, porphyrins have wide practical applications. However, up till now, their bio-based production remains challenging. In this study, we identified potential metabolic factors limiting the biosynthesis of type-III stereoisomeric porphyrins in *Escherichia coli*. To alleviate this limitation, we developed bioprocessing strategies by redirecting more dissimilated carbon flux toward the HemD-enzymatic pathway to enhance the production of type-III uroporphyrin (UP-III), which is a key precursor for heme biosynthesis. Our approaches included the use of antioxidant reagents and strain engineering. Supplementation with ascorbic acid (up to 1 g/L) increased the UP-III/UP-I ratio from 0.62 to 2.57. On the other hand, overexpression of ROS-scavenging genes such as *sod-* and *kat*-genes significantly enhanced UP production in *E. coli*. Notably, overexpression of *sodA* alone led to a 72.9% increase in total porphyrin production (1.56 g/L) while improving the UP-III/UP-I ratio to 1.94. Our findings highlight the potential of both antioxidant supplementation and strain engineering to mitigate ROS-induced oxidative stress and redirect more dissimilated carbon flux toward the biosynthesis of type-III porphyrins in *E. coli*. This work offers an effective platform to enhance the bio-based production of porphyrins.

## 1. Introduction

**Figure 1:**
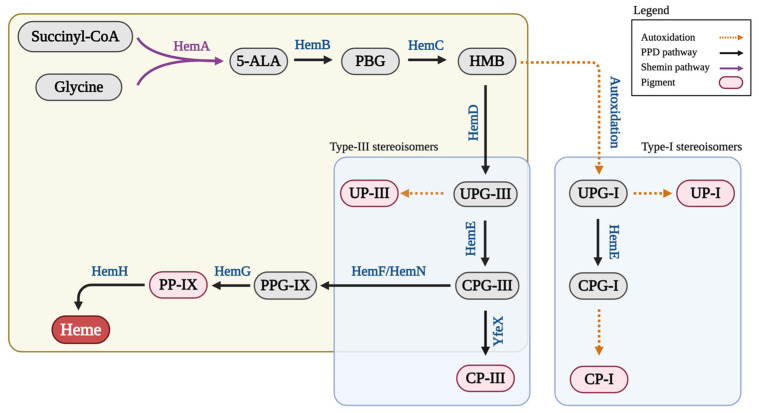
The Shemin/C4 pathway for porphyrin biosynthesis from succinyl-CoA and glycine. 5-ALA, 5-aminolevulinic acid; CPG-I, coproporphyrinogen I; CPG-III, coproporphyrinogen III; CP-I, coproporphyrin I; CP-III, coproporphyrin III; HemA, 5-aminolevulinate synthase; HemB, porphobilinogen synthase; HemC, porphobilinogen deaminase; HemD, uroporphyrinogen III synthase; HemE, uroporphyrinogen decarboxylase; HemF, coproporphyrinogen III oxidase; HemG, protoporphyrinogen oxidase; HemH, protoporphyrin ferrochelatase; HemN, oxygen-independent coproporphyrinogen III oxidase; HMB, Hydroxymethylbilane; PBG, porphobilinogen; PP-IX, protoporphyrin IX; PPG-IX, protoporphyrinogen IX; UP-I, uroporphyrin I; UP-III, uroporphyrin III; UPG-I, uroporphyrinogen I; UPG-III, uroporphyrinogen III; YfeX, porphyrinogen peroxidase.

Porphyrins are essential tetrapyrrolic compounds that serve as precursors for the biosynthesis of heme, chlorophyll, and other biologically critical molecules [1,2,3]. In the Shemin/C4 pathway, which is primarily found in mammals, yeast, fungi, and α-proteobacteria [4], porphyrin biosynthesis begins with the condensation of glycine and succinyl-CoA by 5-aminolevulinic acid synthase (HemA) [5,6,7] (Figure 1). Then, two molecules of 5-aminolevulinic acid (5-ALA) are fused by porphobilinogen synthase (HemB) to form porphobilinogen (PBG) [8]. Four PBG units are subsequently polymerized by porphobilinogen deaminase (HemC) to generate hydroxymethylbilane (HMB), a linear tetrapyrrole [9]. At the HMB branch point, the porphyrin biosynthetic pathway diverges into two distinct routes, leading to the formation of two stereoisomers of uroporphyrinogen I (UPG-I) and uroporphyrinogen III (UPG-III). UPG-I is formed via the spontaneous and non-enzymatic cyclization of HMB [10,11], whereas UPG-III is synthesized enzymatically via uroporphyrinogen III synthase (HemD) [12,13]. Both uroporphyrinogen stereoisomers can undergo further oxidation to form their respective uroporphyrins, i.e., uroporphyrin I (UP-I) and uroporphyrin III (UP-III) [14]. However, the metabolic fates of these stereoisomers differ significantly. In the non-enzymatic branch, UPG-I is converted to coproporphyrinogen I (CPG-I) in a reaction involving uroporphyrinogen decarboxylase (HemE) [15] and is subsequently oxidized to coproporphyrin I (CP-I). CP-I is a metabolic dead-end and cannot be further processed, making it the final product of type-I sterioisomers [14]. In contrast, UPG-III enters the enzymatic branch and undergoes a series of reactions catalyzed by HemE, HemF, HemG, and HemH, ultimately leading to the formation of heme [16,17] (Figure 1).

We recently reported on strain engineering of bacterial *Escherichia coli* for porphyrin production via the Shemin/C4 pathway [18,19]. A metabolic strategy adapted for strain engineering in these studies was the redirection of dissimilated carbon flux toward succinyl-CoA in the citric acid cycle by introducing two mutations of *iclR* and *sdhA*, such that engineered strains with these two mutations can be cultivated aerobically for biosynthesis of succinyl-CoA-derived compounds. However, bacterial cultivation under aerobic conditions might somehow favor the auto-oxidation branch at the metabolic node of HMB, limiting the flux into the HemD-enzymatic branch and the subsequent biosynthesis of type-III stereoisomeric species [18,20]. In fact, cultivation of the engineered *E. coli* strain for UP biosynthesis led to the production of a stereoisomeric mixture with UP-I being dominant. Therefore, redirecting more dissimilated carbon flux toward the HemD-enzymatic branch is highly desirable to enhance the biosynthesis of type-III porphyrins (i.e., UP-III and CP-III) and even heme.

The auto-oxidation at the HMB node is a non-enzymatic process potentially intensified by the presence of reactive oxygen species (ROS) such as superoxide anions, hydrogen peroxide, and hydroxyl radicals [21] which are commonly produced by cells during cellular metabolism under aerobic conditions [22]. Since ROS favor the oxidation of pathway intermediates, their formation can potentially shift more dissimilated carbon flux toward the type I branch and, therefore, the production of UPG-I over UPG-III. This imbalance caused by ROS highlights the critical need to regulate intracellular oxidative stress and, therefore, ensure a more favorable porphyrin metabolism and flux channeling into the pathway toward type-III stereoisomeric species.

ROS are an unavoidable byproduct of aerobic metabolism, particularly under the conditions of high agitation and/or aerobiosis during cultivation [23]. Lowering the aeration in microbial cultures can reduce dissolved oxygen levels and, consequently, the generation of ROS. An alternative strategy to mitigate oxidative stress involves the use of antioxidants, either enzymatic or non-enzymatic ones. Antioxidants are compounds that can delay or inhibit oxidative processes by neutralizing ROS or preventing their formation [24]. Non-enzymatic antioxidants, such as ascorbic acid (vitamin C), are particularly effective due to their dual role as ROS scavengers and stimulators of enzymatic antioxidants [25]. Ascorbic acid, a water-soluble and cost-effective compound, neutralizes ROS by forming ascorbyl radicals, preventing the formation of secondary free-radical byproducts [26]. On the other hand, enzymatic antioxidants, including superoxide dismutases (SODs) and catalases (KATs), play a pivotal role in maintaining redox balance in *E. coli* [27]. SODs act as the first line of defense by converting superoxide anions (O_2_^˙−^) into hydrogen peroxide (H_2_O_2_) and molecular oxygen (O_2_) (Equation (1)), reducing the potential for oxidative damage caused by superoxide radicals [28]. In *E. coli*, three distinct SOD enzymes are encoded, i.e., SodA (Mn-SOD), SodB (Fe-SOD), and SodC (Cu, Zn-SOD) [29]. SodA and SodB function in the cytoplasm, where they neutralize intracellular superoxide [30,31], whereas SodC operates in the periplasm to manage oxidative stress in that compartment [32]. Among these, SodA has been shown to be particularly effective in preventing DNA damage [33].

2 O_2_^˙−^ + 2 H^+^ → H_2_O_2_ + O_2_ (1)

Catalases complement the action of SODs by decomposing hydrogen peroxide, a byproduct of SOD activity, into water and oxygen, thereby preventing its accumulation (Equation (2)) [34]. *E. coli* has two cytoplasmic catalases: KatE, a monofunctional catalase expressed during the stationary phase, and KatG, a bifunctional catalase–peroxidase active during exponential growth [35,36,37,38]. The coordinated activity of these enzymes ensures that ROS levels remain tightly controlled, with superoxide anions concentrations maintained below 0.1 nM and hydrogen peroxide levels below 20 nM [39,40]. Overexpression of both SODs and catalases has been shown to enhance oxidative stress resistance, providing a robust defense system that protects cellular components and stabilizes key biosynthetic processes.

2 H_2_O_2_ → H_2_O + 2 O_2_ (2)

In this study, we explored both antioxidant supplementation and overexpression of ROS-scavenging enzymes to mitigate the effects of oxidative stress and enhance the production of type III porphyrins in *E. coli*. By implementing these bioprocessing strategies, we aim to develop a more efficient and sustainable platform for the bio-based production of porphyrins and their derivatives, as well as address key challenges associated with oxidative stress and metabolic pathway manipulation.

## 2. Materials and Methods

### 2.1. Bacterial Strains and Plasmids

Details of the bacterial strains and plasmids used in this study are provided in Table 1, while the primer sequences are listed in Table S1. Taq DNA polymerase was sourced from New England Biolabs (Ipswich, MA, USA). Genomic DNA extraction from bacterial cells was carried out using the Qiagen Blood & Tissue DNA Isolation Kit (Hilden, Germany), and plasmid purification was performed using the Qiagen Miniprep kit. The CPC-Sbm-derived host cells used in this study were developed from the CPC-Sbm strain, which carries targeted mutations in the *sdhA* and/or *iclR* genes [41]. This strain originates from the *E. coli* BW25113 background, where the ldhA gene had been previously inactivated [42]. For molecular cloning purposes, *E. coli* HI-Control 10G (Lucigen, Middleton, WI, USA) was employed. Plasmid construction was carried out using the Gibson assembly method [43], and DNA sequencing services were provided by Plasmidsaurus (Eugene, OR, USA). Oligonucleotide synthesis was conducted by Integrated DNA Technologies (IDT) (Coralville, IA, USA).

Plasmids pK-hemA, pK-hemAB, pK-hemABC, pK-hemABCD, and pK-hemAB-E were used from our previous studies [18,19].

pK-hemABsodABC was constructed by amplifying *hemAB* and amplifying the backbone using primer sets P1/P2 and P3/P4 with pK-hemABCD as the template. The *sodA*, *sodB*, and *sodC* genes were amplified from the genomic DNA of *E. coli* MG1655 using primer sets P5/P6, P7/P8, and P9/P10. These five fragments were Gibson assembled to form pK-hemABsodABC. For effective coexpression, all genes were aligned to form an operon *hemABsodABC* regulated by a common strong *trc* promoter with an individual, strong RBS for each gene.

pK-hemABsodA was constructed by amplifying *hemAB* and amplifying the backbone using primer sets P1/P2 and P11/P4 with pK-hemABCD as the template. The *sodA* gene was amplified from the genomic DNA of *E. coli* MG1655 using primers P5/P12. These three fragments were Gibson assembled to form pK-hemABsodA with all genes arranged on a single operon regulated by a common strong *trc* promoter.

Similarly, pK-hemABsodB was constructed by amplifying *hemAB* and amplifying the backbone using primer sets P1/P2 and P13/P4 with pK-hemABCD as the template. The *sodB* gene was amplified from the genomic DNA of *E. coli* MG1655 using primers P14/P15. These three fragments were Gibson assembled to form pK-hemABsodB with all genes arranged on a single operon regulated by a common strong *trc* promoter.

pK-hemABsodC was constructed by amplifying *hemAB* and amplifying the backbone using primer sets P1/P2 and P3/P4 with pK-hemABCD as the template. The *sodC* gene was amplified from the genomic DNA of *E. coli* MG1655 using primers P16/P10. These three fragments were Gibson assembled to form pK-hemABsodB with all genes arranged on a single operon regulated by a common strong *trc* promoter.

pK-hemABsodABC-katEG was constructed by amplifying *hemAB* and amplifying the backbone using primer sets P1/P2 and P17/P4 with pK-hemABCD as the template. The *sodABC* genes were amplified from pK-hemABsodABC using the primers P18/P19. The *grac*max promoter was amplified from pK-hemAB-E using the primers P20/P21. The *katE* and *katG* genes were amplified from the genomic DNA of *E. coli* MG1655 using primer sets P22/P23 and P24/P25. These six fragments were Gibson assembled to form pK-hemABsodABC-katEG, and for effective expression, *hemABsodABC* were arranged on the first operon regulated by a common strong *trc* promoter, and *katEG* was arranged on the second operon regulated by the strong *grac*max promoter.

pK-hemAB-katE was constructed by amplifying *hemAB* along with the *grac*max promoter and amplifying the backbone using primer sets P26/P21 and P27/P4 and pK-hemAB-E as the template. The *katE* gene was amplified from the genomic DNA of *E. coli* MG1655 using primers P22/P28. These three fragments were Gibson assembled to form pK-hemAB-katE with *hemAB* arranged on the first operon regulated by a common strong *trc* promoter and *katE* on the second operon regulated by the strong *grac*max promoter.

Similarly, pK-hemAB-katG was constructed by amplifying *hemAB* along with the *grac*max promoter and amplifying the backbone using primer sets P26/P29 and P17/P4 with pK-hemAB-E as the template. The *katG* gene was amplified from the genomic DNA of *E. coli* MG1655 using primers P30/P25. These three fragments were Gibson assembled to form pK-hemAB-katG with *hemAB* arranged on the first operon regulated by a common strong *trc* promoter and *katG* on the second operon regulated by the strong *grac*max promoter.

pK-hemABsodA-katE was constructed by amplifying *hemABsodA* and amplifying the backbone using primer sets P26/P31 and P27/P4 with pK-hemABsodA as the template. The *grac*max promoter along with the *katE* gene were amplified from pK-hemABsodABC-katEG using the primers P32/P28. These three fragments were Gibson assembled to form pK-hemABsodA-katE with *hemABsodA* arranged on the first operon regulated by a common strong *trc* promoter and *katE* on the second operon regulated by the strong *grac*max promoter.

Similarly, pK-hemABsodA-katG was constructed by amplifying *hemABsodA* and amplifying the backbone using primer sets P26/P31 and P17/P4 with pK-hemABsodA as the template. The *grac*max promoter was amplified from pK-hemABsodABC-katEG using the primers P32/P29. The *katG* gene was amplified from the genomic DNA of *E. coli* MG1655 using primers P30/P25. These four fragments were Gibson assembled to form pK-hemABsodA-katG with *hemABsodA* arranged on the first operon regulated by a common strong *trc* promoter and *katG* on the second operon regulated by the strong *grac*max promoter.

### 2.2. Media and Bacterial Cell Cultivation

All media components were obtained from Sigma-Aldrich Co. (St. Louis, MO, USA), except for yeast extract and tryptone, which were obtained from BD Diagnostic Systems (Franklin Lakes, NJ, USA). *E. coli* strains were stored as glycerol stocks at −80 °C and streaked onto lysogeny broth (LB) agar plates, prepared with 10 g/L tryptone, 5 g/L yeast extract, and 5 g/L NaCl. Plates were incubated at 37 °C for 14–16 h. For bioreactor experiments, single colonies were picked from LB agar plates and inoculated into 12 mL of super broth (SB) medium (32 g/L tryptone, 20 g/L yeast extract, and 5 g/L NaCl) in a 125 mL Erlenmeyer flask. This culture was grown at 37 °C and 280 rpm on a rotary shaker (New Brunswick Scientific, Edison Township, NJ, USA) for 4–6 h and was then used to inoculate 220 mL of SB medium at 2% (*v*/*v*) in a 1 L conical flask. The seed culture was incubated overnight (14–16 h) under the same conditions. Cells were harvested by centrifugation at 4500× *g* and 20 °C for 8 min and then resuspended in 40 mL of fresh SB medium. The resuspended culture was used to inoculate a stirred tank bioreactor (CelliGen 115, Eppendorf AG) containing 0.8 L of working volume, set at 37 °C and stirred at 430 rpm. The semi-defined medium used in the batch bioreactor consisted of 30 g/L glycerol, 0.23 g/L K_2_HPO_4_, 0.51 g/L NH_4_Cl, 49.8 mg/L MgCl_2_, 48.1 mg/L K_2_SO_4_, 1.52 mg/L FeSO_4_, 0.055 mg/L CaCl_2_, 2.93 g/L NaCl, 0.72 g/L tricine, 10 g/L yeast extract, 10 mM NaHCO_3_, and 1 mL/L trace elements (2.86 g/L H_3_BO_3_, 1.81 g/L MnCl_2_·4H_2_O, 0.222 g/L ZnSO_4_·7H_2_O, 0.39 g/L Na_2_MoO_4_·2H_2_O, 79 μg/L CuSO_4_·5H_2_O, and 49.4 μg/L Co(NO_3_)_2_·6H_2_O) [44]. To induce protein expression, 0.05 mM isopropyl β-D-1-thiogalactopyranoside (IPTG) was added. To prevent glycine limitation during cultivation, 2 g of glycine was supplemented into the bioreactor approximately 30 h after inoculation. For certain batches, ascorbic acid was included in the medium at concentrations between 0.5 and 2 g/L. Aerobic conditions were maintained by purging air into the bioreactor at a rate of 1 volume of air per volume of liquid per minute (vvm). The pH of the culture was controlled at 7.0 ± 0.1 by automatic addition of 3 M NH_4_OH or 3 M H_3_PO_4_.

### 2.3. Analysis

Cell density at OD_600_ was measured by washing all culture samples once with a 0.15 M saline solution, diluting them appropriately, and analyzing them using a GENESYSTM 40/50 Vis/UV-Vis spectrophotometer (Thermo Fisher Scientific, Waltham, MA, USA). For the preparation of cell-free medium, culture samples were centrifuged at 17,000× *g* for 1 min and then filtered through a 0.2 μm syringe filter to ensure sterility. High-performance liquid chromatography (HPLC) was used to analyze extracellular metabolites and glycerol, employing a Shimadzu LC-10AT system (Shimadzu, Kyoto, Japan) with a refractive index detector (RID-10A, Shimadzu) and an Aminex HPX-87H chromatographic column (Bio-Rad Laboratories, Hercules, CA, USA). The column temperature was maintained at 35 °C, and the mobile phase consisted of 5 mM H_2_SO_4_ (pH 2), running at a flow rate of 0.6 mL/min. Data from the RID were processed using Clarity Lite software (v. 7.4.1.88, DataApex, Prague, Czech Republic).

Levels of 5-ALA and PBG in the cell-free medium were quantified using a modified Ehrlich’s reagent assay [45]. Porphyrins were analyzed using a Waters™ 2690 separation module equipped with a photodiode array (PDA) detector (2996 PDA detector, Waters™, Milford, MA, USA) and a Chromolith^®^ HighResolution RP-18 endcapped column (Supelco, Darmstadt, Germany). UV absorbance was detected at 400 nm, and data were processed using Empower 3 software (Waters™, Milford, MA, USA). The mobile phase system used for porphyrin analysis followed a previously published method [9] with minor modifications.

The percentage yield of UP was determined by calculating the molar ratio of the produced UP to the theoretical maximum production, based on the amount of glycerol consumed. Statistical analyses were performed to compare the UP-III/UP-I ratio under different conditions, and the detailed results are provided in the Supplementary Materials.

## 3. Results

### 3.1. Effects of Ascorbic Acid Supplementation on UP Biosynthesis

To evaluate the effects of antioxidant supplementation on limiting flux toward the auto-oxidation pathway, we utilized DMB, a UP-producing strain heterologously co-expressing *hemA* from *R. sphaeroides* and *hemB* from *E. coli*, as described in our previous study [18]. Cultivation of DMB was conducted with ascorbic acid at 0, 0.5, 1, and 2 g/L, respectively.

**Figure 2:**
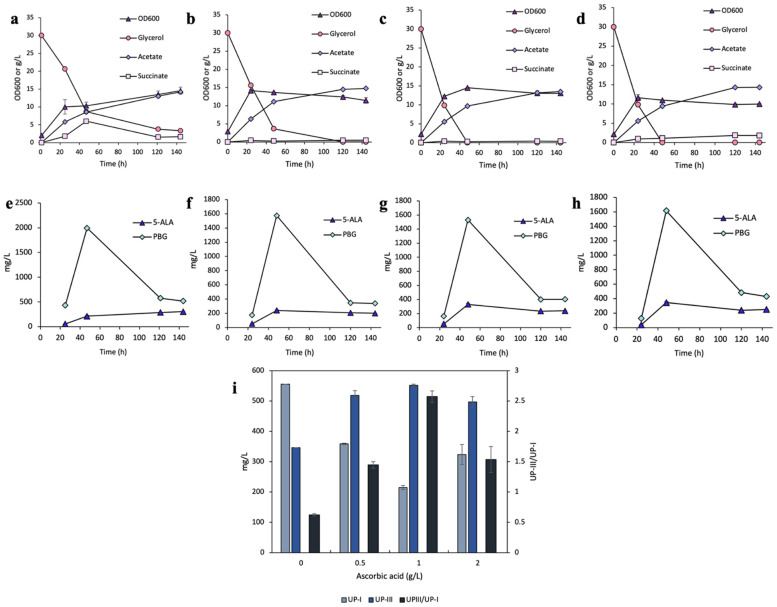
Aerobic bioreactor cultivation of DMB for UP biosynthesis with ascorbic acid supplementation. Time profiles of (**a**–**d**) cell density (OD_600_), glycerol consumption, and acetate/succinate formation, (**e**–**h**) 5-ALA and PBG biosynthesis, and (**i**) UP biosynthesis at 144 h. All values are reported as means ± SD (n = 2).

Enhanced glycerol consumption was observed with ascorbic acid supplementation (Figure 2a–d). Maximum cell density (~14.4 OD_600_) was observed with ascorbic acid concentrations ranging from 0 to 1 g/L, whereas increasing the concentration to 2 g/L slightly reduced the maximum cell density to 12.2 OD_600_. Acetate profile followed a similar increasing trend across all culture conditions, with final titers reaching ~14.5 g/L by the end of cultivation. In contrast, compared to the control, the succinate accumulation profile was significantly reduced in cultivations supplemented with ascorbic acid.

Time profiles of 5-ALA and PBG showed a similar trend with peaks occurring at 48 h across all conditions (Figure 2e–h). However, cultivations supplemented with ascorbic acid yielded slightly lower PBG titers of ~1600 mg/L, compared to 1995 mg/L (193 mg/OD_600_/L) for the control cultivation without ascorbic acid.

While the total UP production was slightly reduced with the addition of ascorbic acid, the UP distribution was more favorable toward UP-III with the UP-III/UP-I ratios of 0.6, 1.4, 2.6, and 1.5 for cultivations with 0, 0.5, 1, and 2 g/L of ascorbic acid, respectively (Figure 2i). Notably, cultivation with 1 g/L ascorbic acid had 215 mg/L (1.3% yield) UP-I and 552 mg/L (3.3% yield) UP-III, indicating a significant shift in dissimilated carbon flux toward the enzymatic (HemD) pathway. However, increasing the ascorbic acid concentration beyond 1 g/L did not further increase the UP-III/UP-I ratio but led to reduced cell growth.

### 3.2. Effects of SODs on UP Biosynthesis

To assess the effects of SODs in redirecting dissimilated carbon flux toward the enzymatic (HemD) pathway, we engineered a UP-producing strain, SOD1, by heterologously co-expressing *hemA* from *R. sphaeroides* along with *hemB* and *sodABC* from *E. coli*. These genes were arranged in a single operon under the control of a strong *trc* promoter, with each gene translationally regulated by a strong RBS. Unexpectedly, this strain did not exhibit any pigmentation, suggesting that the overexpression of *sodABC* might somehow interfere with porphyrin production. To identify the specific gene(s) responsible for the interference, we constructed three additional strains, each co-expressing *hemAB* and an individual *sod* gene, i.e., *sodA* (SOD2), *sodB* (SOD3), or *sodC* (SOD4). Notably, SOD4 showed no pigmentation, indicating that the overexpression of *sodC* was likely responsible for interfering with porphyrin production.

**Figure 3:**
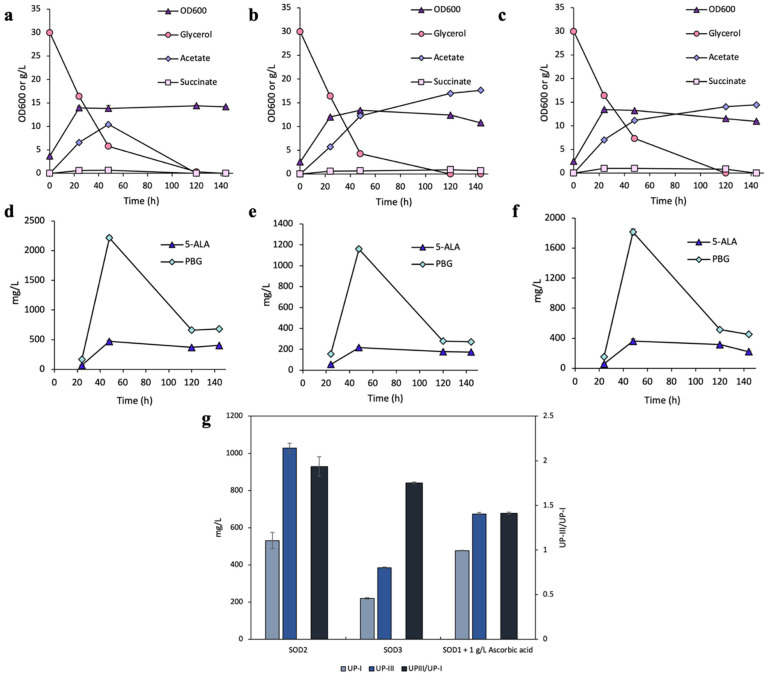
Aerobic bioreactor cultivation of SOD2 and SOD3 for UP biosynthesis with ascorbic acid supplementation. Time profiles of (**a**–**c**) cell density (OD_600_), glycerol consumption, and acetate/succinate formation, (**d**–**f**) 5-ALA and PBG biosynthesis, and (**g**) UP biosynthesis at 144 h. All values are reported as means ± SD (n = 2).

Both strains of SOD2 and SOD3 exhibited a similar and slow glycerol consumption pattern, and glycerol was completely depleted by 120 h (Figure 3a,b). Cell density for SOD2 was 14.4 OD_600_ at 120 h, while SOD3 reached 13.4 OD_600_ at 48 h. SOD2 produced 10.4 g/L acetate within the first 48 h, followed by diauxic growth with the accumulated acetate being depleted by the end of the cultivation. On the other hand, SOD3 exhibited consistent acetate accumulation throughout the cultivation, reaching 17.7 g/L by 120 h. Both strains showed minimal succinate production.

The time profiles of 5-ALA and PBG for SOD2 and SOD3 displayed a similar trend, with titers peaking at 48 h (Figure 3c,d). 5-ALA titers reached 469 and 217 mg/L (33.9 and 16.1 mg/OD_600_/L), while PBG titers reached 2215 mg/L and 316 mg/L (160 and 86.4 mg/OD_600_/L) in SOD2 and SOD3, respectively.

The final UP production at 144 h for SOD2 was 531 mg/L (3.1% yield) UP-I and 1029 mg/L (6.1% yield) UP-III, respectively, achieving a UP-III/UP-I ratio of 1.9 (Figure 3g). This represented a significant shift in dissimilated carbon flux from the auto-oxidation pathway to the enzymatic (HemD) pathway compared to the control DMB strain. Additionally, SOD2 achieved a high total UP titer of 1.56 g/L (9.2% yield), 72.9% higher than the control DMB strain. On the other hand, SOD3 produced 219.9 mg/L (1.3% yield) UP-I and 385.3 mg/L (2.3% yield) of UP-III, with a UP-III/UP-I ratio of 1.8 (Figure 3g). While SOD3 had a high UP-IIII/UP-I ratio similar to SOD2, the total UP titer was significantly lower than SOD2.

In another experiment, we combined the above non-enzymatic and enzymatic strategies by supplementing 1 g/L ascorbic acid upon cultivating SOD2. The resulting cultivation had a slightly reduced cell density and slower glycerol consumption (Figure 3c) compared to the control SOD2 cultivation without ascorbic acid. Additionally, this strain exhibited significant acetogenesis with the acetate titer reaching 14.4 g/L by the end of cultivation, suggesting that ascorbic acid suppressed the diauxic growth observed in the control SOD2 cultivation. Time profiles for 5-ALA and PBG had peaks of 361 mg/L (27.2 mg/OD_600_/L) and 1814 mg/L (137 mg/OD_600_/L), respectively (Figure 3d), both lower than those in the control SOD2 cultivation. This cultivation produced 477 mg/L (2.8% yield) UP-I and 674 mg/L (4.0% yield) UP-III, with a UP-III/UP-I ratio of 1.4 (Figure 3g). These results suggest that there were no combined or synergistic effects for the two enzymatic or non-enzymatic strategies, although the possibility of such interactions cannot be ruled out without further analysis.

### 3.3. Effects of Catalases on UP Biosynthesis

To investigate the effects on UP biosynthesis, we initially constructed a KAT1 strain, which contains the first operon for co-expressing *hemAB* and *sodABC* under the control of the *trc* promoter and the second operon for co-expressing *katEG* under the control of the *gracmax* promoter. This expression strategy was designed to integrate the superoxide-neutralizing activity of SOD-ABC with the H_2_O_2_-scavenging capacity of KAT-EG. However, KAT1 only produced a limited amount of porphyrin with minimal pigmentation, prompting further investigation into the effects from individual *kat* genes. Hence, we constructed the strains KAT2 and KAT3, which expressed *katE* and *katG* in the plasmids pK-HemAB-KatE and pK-HemAB-KatG, respectively. To investigate the combined effects of *kat* and *sodA* genes, we generated additional strains, KAT4 and KAT5, harboring the plasmids pK-HemABSodA-KatE and pK-HemABSodA-KatG, respectively.

**Figure 4:**
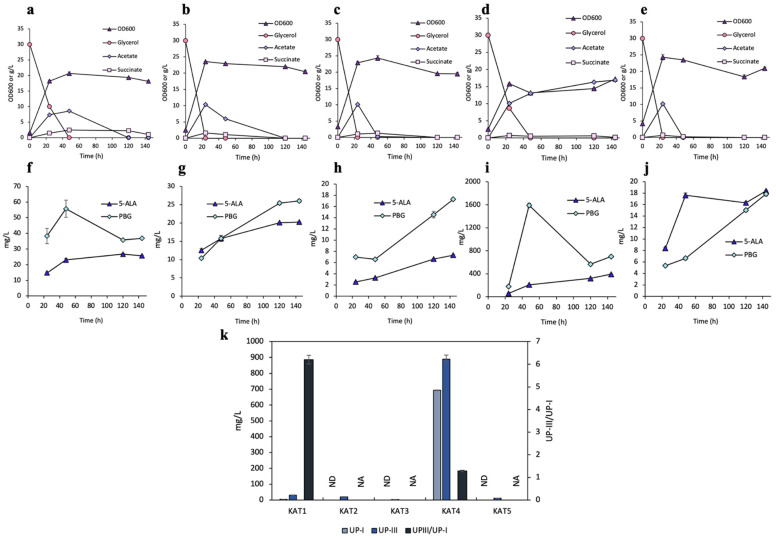
Aerobic bioreactor cultivation of KAT1, KAT2, KAT3, KAT4, and KAT5 for UP biosynthesis. Time profiles of (**a**–**e**) cell density (OD_600_), glycerol consumption, and acetate/succinate formation, (**f**–**j**) 5-ALA and PBG biosynthesis, and (**k**) UP biosynthesis at 144 h. All values are reported as means ± SD (n = 2).

All KAT strains, except KAT4, had enhanced cell growth, achieving maximum cell densities between 20 and 25 OD_600_, with distinct diauxic growth (Figure 4a–e). KAT4 had a lower cell density of 17.2 OD_600_, with notable acetogenesis. Minimal 5-ALA and PBG were produced across all strains except KAT4 (Figure 4f–j). Specifically, KAT4 produced 393 mg/L (22.8 mg/OD_600_/L) 5-ALA at 144 h and 1591 mg/L (121 mg/OD_600_/L) PBG at 48 h (Figure 4i).

Unexpectedly, KAT2, KAT3, and KAT5 produced negligible UP, with minimal UP-I being detected (Figure 4k). While KAT1 had a remarkable UP-III/UP-I ratio of 6.2, its total UP production was less than 40 mg/L. On the other hand, KAT4 had substantial UP production comparable to SOD2, with a high total UP titer of 1.58 g/L, consisting of 693 mg/L (4.1% yield) UP-I and 890 mg/L (5.3% yield) UP-III, and a decent UP-III/UP-I ratio of 1.3.

## 4. Discussion

In our previous study [18], we demonstrated that while HemABCD are the enzymes involved in UP biosynthesis, overexpression of *hemAB* alone was sufficient for effective UP biosynthesis. However, the majority of the produced UP was the stereoisomer UP-I rather than UP-III [18], primarily due to the more effective auto-oxidation pathway rather than the HemD-enzymatic pathway at the metabolic node of HMB (Figure 1) under the aerobic cultivation conditions for porphyrin biosynthesis [21]. In this study, we explored redirecting the dissimilated carbon flux more toward the HemD-enzymatic pathway to favor UP-III production while limiting the auto-oxidation pathway through a combination of bioprocessing and genetic strategies, specifically including antioxidant supplementation and strain engineering for ROS-scavenging enzyme overexpression.

We began by investigating the effects of antioxidant supplementation using ascorbic acid, which is a natural, water-soluble, and cost-effective compound known for its ROS-scavenging properties [46]. A range of concentrations (0–2 g/L) of ascorbic acid was supplemented upon cultivation to evaluate its influence on UP biosynthesis in *E. coli*. Our results revealed that supplementing ascorbic acid up to 1 g/L significantly enhanced the UP-III/UP-I ratio while maintaining approximately the same level of total UP biosynthesis, indicating a successful redirection of the dissimilated carbon flux toward the HemD-enzymatic pathway. However, increasing the concentration to 2 g/L did not yield further improvement in flux redirection, but, in fact, led to a reduction in total UP biosynthesis and a noticeable retardation of cell growth. This inhibitory effect at higher ascorbic acid concentrations aligns with previous studies, which reported that ascorbic acid could exhibit antibacterial properties in the range of 2–20 g/L [47,48]. Ascorbic acid facilitates intracellular redox homeostasis by directly scavenging ROS, thereby reducing oxidative stress and protecting key biosynthetic enzymes from damage [49]. By lowering ROS levels, ascorbic acid minimizes the diversion of intermediates toward the non-enzymatic auto-oxidation pathway, allowing metabolic flux to be redirected toward the HemD-enzymatic pathway. This redirection enhances type-III porphyrin production while reducing the accumulation of type-I porphyrins. These observations highlight the importance of adjusting antioxidant levels to achieve a physiological balance between ROS mitigation and maintaining cellular viability and metabolic activity during cell cultivation.

Next, we explored the use of enzymatic antioxidants by overexpressing various *sod* genes in UP-producing *E. coli* strains. Among these *sod* genes, *sodA* appeared to be the most effective one in redirecting the dissimilated carbon flux toward the HemD-enzymatic pathway. Notably, the UP-producing strain with *sodA* overexpression not only had a high UP-III/UP-I ratio of 1.9 but also enhanced total UP production to 1.56 g/L, which was 72.9% more than the control cultivation. The observed improvement in UP biosynthesis could be attributed to the role of SodA in mitigating oxidative stress, which protects key enzymes in the biosynthetic pathway from damage caused by ROS. Overexpression of *sodA* has been shown in multiple studies to enhance product titers such as 5-ALA [50,51]. Notably, one study reported that the improved redox balance achieved by *sod* gene overexpression enhanced the activity of key enzymes directly involved in product formation, a mechanism that may also explain the improved yields observed in our study [52]. In addition, the ability of SodA to balance intracellular redox conditions may indirectly shift more dissimilated carbon flux away from auto-oxidation for UP-III biosynthesis. The function of SodA in protecting enzymatic activities under oxidative stress has been reported, as its inactivation led to elevated oxidation of specific enzymes and reduced ATP/NADH levels [53].

Additionally, while overexpression of *sodB* also resulted in a relatively high UP-III/UP-I ratio, the total UP biosynthesis was significantly reduced compared to overexpression of *sodA*. This may be due to differences in the catalytic efficiency and metal-ion-dependency of Sod enzymes, with Mn-dependent SodA likely providing more effective oxidative stress mitigation under the physiological conditions for UP biosynthesis. Conversely, *sodC* overexpression somehow disrupted the cell’s metabolic activity for porphyrin production, and the cultivation had no pigmentation. This effect may be associated with the periplasmic localization of SodC, limiting its activity to the periplasm as superoxide cannot cross cellular membranes [54]. The confined activity of SodC could result in an imbalance in redox homeostasis between the periplasm and cytoplasm, potentially interfering with select intracellular processes critical for porphyrin biosynthesis. Additionally, the activity of SodC may contribute to hydrogen peroxide accumulation in the periplasm, a phenomenon reported in studies associating *sodC* overexpression with altered redox dynamics and dye degradation [55].

Combining the two strategies of ascorbic acid supplementation and *sodA* overexpression did not show clear evidence of synergistic effects. Instead, the results suggest that relying on either enzymatic (i.e., SodA) or non-enzymatic (i.e., ascorbic acid) antioxidants alone may be more beneficial for redirecting dissimilated carbon flux upon UP biosynthesis. This could be attributed to potential redundancy or competition in their antioxidative mechanisms, indicating the need for a balanced approach tailored to specific oxidative conditions. However, further studies are needed to fully understand the relationship between their antioxidative mechanisms.

Overexpression of SOD genes can effectively scavenge superoxide radicals and convert them into hydrogen peroxide, which plays a dual role in cellular metabolism. While low-level hydrogen peroxide acts as a signaling molecule essential for various cellular processes, including heme biosynthesis [56,57] and catalase gene (*katG*) induction [34,58], excessive concentration of hydrogen peroxide might be toxic to the cells by exhibiting adverse effects due to DNA damage [59,60]. Therefore, regulating the hydrogen peroxide level is critical for maintaining cellular homeostasis for optimal physiological and metabolic activities. To achieve this balance in the intracellular level of hydrogen peroxide, we explored co-overexpression of the *sod* genes with various catalase genes by deriving strains KAT1-KAT5. KAT1, expressing both *sodABC* and *katEG* along with *hemAB*, appears to disrupt porphyrin biosynthesis by exhibiting minimal pigmentation, prompting us for a closer examination of individual *kat* genes. Strains KAT2 and KAT3, overexpressing *katE* and *katG*, respectively, along with *hemAB*, also exhibited negligible porphyrin production. This could be attributed to excessive degradation of the minimal-level hydrogen peroxide required for triggering porphyrin biosynthesis and other cellular processes. As catalase overexpression alone was proved ineffective, individual *katE* and *katG* were subsequently co-expressed with *sodA*, generating the strains KAT4 and KAT5. Among them, KAT4 had superior culture performance, producing 1.58 g/L UP with a UP-III/UP-I ratio of 1.3. These results suggest that the combination of *sodA* and *katE* could not only effectively eliminate superoxides but also balance ROS detoxification, successfully directing more dissimilated carbon flux toward the HemD-enzymatic pathway for enhanced UP-III biosynthesis. The results highlight the importance of the intricate balance required to manage ROS and minimize cellular stress, both contributing toward UP-III biosynthesis. Hence, identification of alternative antioxidant systems and their interactions for genetic modification may provide new avenues to enhance porphyrin biosynthesis.

Recent advancements in porphyrin synthesis have demonstrated the applicability of metabolic engineering strategies and pathway regulation in enhancing biosynthetic efficiency [61,62], whereas integrated optimization approaches further highlight the potential for improving biosynthesis [63]. Aligned with these advancements, our study demonstrates that cost-effective antioxidant supplementation, such as ascorbic acid, combined with the overexpression of ROS-scavenging enzymes (e.g., SodA and KatE), significantly enhances porphyrin biosynthesis and highlights its potential for industrial-scale applications.

## Figures and Tables

**Table 1:** Strains and plasmids used in this study.

Name	Description or Relevant Genotype	Source
Host strains		
HI-Control 10G	*mcrA*, ∆(*mrr-hsdRMS-mcrBC*), *endA1*, *recA1*, *ϕ*80*dlacZ*∆M15, ∆*lacX74*, *araD139*, ∆(*ara leu*)*7697*, *galU*, *galK*, *rpsL* (Str^R^), *nupG*, *λ^−^*, *tonA*, Mini-F *lacI*^q1^ (Gent^R^)	Lucigen
MG1655	K-12; F^−^, *λ*^−^, *rph-1*	Lab stock
CPC-Sbm∆*iclR*∆*sdhA*	F^−^, ∆*(araD-araB)567*, ∆*lacZ4787(::rrnB-3)*, *λ*^−^, *rph-1*, ∆*(rhaD-rhaB)568*, *hsdR514*, ∆*ldhA*, P*_trc_::sbm* (i.e., with the FRT-P*_trc_*cassette replacing the 204-bp upstream of the Sbm operon), ∆*iclR*, ∆*sdhA*	[41]
DMB	CPC-Sbm∆*iclR*∆*sdhA*/pK-hemAB	[18]
SOD1	CPC-Sbm∆*iclR*∆*sdhA*/pK-hemABsodABC	This study
SOD2	CPC-Sbm∆*iclR*∆*sdhA*/pK-hemABsodA	This study
SOD3	CPC-Sbm∆*iclR*∆*sdhA*/pK-hemABsodB	This study
SOD4	CPC-Sbm∆*iclR*∆*sdhA*/pK-hemABsodC	This study
KAT1	CPC-Sbm∆*iclR*∆*sdhA*/pK-hemABsodABC-katEG	This study
KAT2	CPC-Sbm∆*iclR*∆*sdhA*/pK-hemAB-katE	This study
KAT3	CPC-Sbm∆*iclR*∆*sdhA*/pK-hemAB-katG	This study
KAT4	CPC-Sbm∆*iclR*∆*sdhA*/pK-hemABsodA-katE	This study
KAT5	CPC-Sbm∆*iclR*∆*sdhA*/pK-hemABsodA-katG	This study
Plasmids		
pK-hemA	p15A ori, Km^R^, P*_trc_::hemA*	[18]
pK-hemAB	p15A ori, Km^R^, P*_trc_::hemAB*	[18]
pK-hemABC	p15A ori, Km^R^, P*_trc_::hemABC*	[18]
pK-hemAB-E	p15A ori, Km^R^, P*_trc_:: hemAB -*P*_grac_*_max_*::hemE*	[19]
pK-hemABsodABC	p15A ori, Km^R^, P*_trc_::hemABsodABC*	This study
pK-hemABsodA	p15A ori, Km^R^, P*_trc_::hemABsodA*	This study
pK-hemABsodB	p15A ori, Km^R^, P*_trc_::hemABsodB*	This study
pK-hemABsodC	p15A ori, Km^R^, P*_trc_::hemABsodC*	This study
pK-hemABsodABC-katEG	p15A ori, Km^R^, P*_trc_:: hemABsodABC-*P*_grac_*_max_*::katEG*	This study
pK-hemAB-katE	p15A ori, Km^R^, P*_trc_:: hemAB-*P*_grac_*_max_*::katE*	This study
pK-hemAB-katG	p15A ori, Km^R^, P*_trc_:: hemAB-*P*_grac_*_max_*::katG*	This study
pK-hemABsodA-katE	p15A ori, Km^R^, P*_trc_:: hemABsodA-*P*_grac_*_max_*::katE*	This study
pK-hemABsodA-katG	p15A ori, Km^R^, P*_trc_:: hemABsodA-*P*_grac_*_max_*::katG*	This study

## Data Availability

The original contributions presented in this study are included in the article/Supplementary Materials. Further inquiries can be directed to the corresponding author.

## Supplementary Materials

The following supporting information can be downloaded at https://www.mdpi.com/article/10.3390/bioengineering12010083/s1: Table S1: Oligomers used in this study. Table S2: Statistical analysis data for the UP-III/UP-I ratio for 0.5 g/L ascorbic acid supplementation vs. the control strain. Table S3: Statistical analysis data for the UP-III/UP-I ratio for 1 g/L ascorbic acid supplementation vs. the control strain. Table S4: Statistical analysis data for the UP-III/UP-I ratio for 2 g/L ascorbic acid supplementation vs. the 0.5 g/L ascorbic acid supplementation. Table S5: Statistical analysis data for the UP-III/UP-I ratio for SOD2 vs. the control strain. Table S6: Statistical analysis data for the UP-III/UP-I ratio for SOD3 vs. the control strain. Table S7: Statistical analysis data for the UP-III/UP-I ratio for SOD2 + 1 g/L ascorbic acid supplementation vs. the control strain. Table S8: Statistical analysis data for the UP-III/UP-I ratio for KAT1 vs. the control strain. Table S9: Statistical analysis data for the UP-III/UP-I ratio for KAT2 vs. the control strain.

## References

[1] Shemin D. (1975). Porphyrin synthesis: Some particular approaches. Ann. N. Y. Acad. Sci..

[2] Mauzerall D.C. (1998). Evolution of porphyrins. Clin. Dermatol..

[3] Schlicke H., Richter A., Rothbart M., Brzezowski P., Hedtke B., Grimm B. (2015). Function of tetrapyrroles, regulation of tetrapyrrole metabolism and methods for analyses of tetrapyrroles. Procedia Chem..

[4] Beale S.I. (2007). Biosynthesis of hemes. EcoSal Plus.

[5] Shemin D. (1970). On the synthesis of heme. Naturwissenschaften.

[6] Kang D.-K., Kim S.-S., Chi W.-J., Hong S.-K., Kim H.-K., KIM H.-U. (2004). Cloning and Expression of the *Rhodobacter capsulatus hemA* Gene in *E. coli* for the Production of S-Aminolevulinic Acid. J. Microbiol. Biotechnol..

[7] Jordan P.M., Shemin D. (1972). 11 δ-Aminolevulinic Acid Synthetase. The Enzymes.

[8] Layer G., Reichelt J., Jahn D., Heinz D.W. (2010). Structure and function of enzymes in heme biosynthesis. Protein Sci..

[9] Kwon S.J., De Boer A.L., Petri R., Schmidt-Dannert C. (2003). High-Level Production of Porphyrins in Metabolically Engineered *Escherichia coli*: Systematic Extension of a Pathway Assembled from Overexpressed Genes Involved in Heme Biosynthesis. Appl. Environ. Microbiol..

[10] Dailey H.A., Dailey T.A., Gerdes S., Jahn D., Jahn M., O’Brian M.R., Warren M.J. (2017). Prokaryotic heme biosynthesis: Multiple pathways to a common essential product. Microbiol. Mol. Biol. Rev..

[11] Mathews M.A., Schubert H.L., Whitby F.G., Alexander K.J., Schadick K., Bergonia H.A., Phillips J.D., Hill C.P. (2001). Crystal structure of human uroporphyrinogen III synthase. EMBO J..

[12] Yang Q., Zhao J., Zheng Y., Chen T., Wang Z. (2023). Microbial synthesis of heme b: Biosynthetic pathways, current strategies, detection, and future prospects. Molecules.

[13] Medlock A.E., Dailey H.A. (2017). Heme Biosynthesis. Met. Act. Site Assem..

[14] Di Pierro E., Brancaleoni V., Granata F. (2016). Advances in understanding the pathogenesis of congenital erythropoietic porphyria. Br. J. Haematol..

[15] Elder G., Roberts A. (1995). Uroporphyrinogen decarboxylase. J. Bioenerg. Biomembr..

[16] Zámocký M., Hofbauer S., Gabler T., Furtmüller P.G. (2023). The molecular evolution, structure, and function of coproporphyrinogen oxidase and protoporphyrinogen oxidase in prokaryotes. Biology.

[17] Aftab H., Donegan R.K. (2024). Regulation of heme biosynthesis via the coproporphyrin dependent pathway in bacteria. Front. Microbiol..

[18] Arab B., Westbrook A.W., Moo-Young M., Liu Y., Chou C.P. (2024). Bio-Based Production of Uroporphyrin in *Escherichia coli*. Synth. Biol. Eng..

[19] Arab B., Westbrook A., Moo-Young M., Liu Y., Chou C.P. (2024). High-Level Bio-Based Production of Coproporphyrin in *Escherichia coli*. Fermentation.

[20] Dailey H.A. (1997). Enzymes of heme biosynthesis. JBIC J. Biol. Inorg. Chem..

[21] Lushchak V.I. (2014). Free radicals, reactive oxygen species, oxidative stress and its classification. Chem. -Biol. Interact..

[22] Ozougwu J.C. (2016). The role of reactive oxygen species and antioxidants in oxidative stress. Int. J. Res..

[23] Lu C., Bentley W.E., Rao G. (2003). Comparisons of oxidative stress response genes in aerobic *Escherichia coli* fermentations. Biotechnol. Bioeng..

[24] Voronkova Y., Voronkova O., Gorban V., Holoborodko K. (2018). Oxidative stress, reactive oxygen species, antioxidants: A review. Ecol. Noospherology.

[25] Gęgotek A., Skrzydlewska E. (2023). Ascorbic acid as antioxidant. Vitam. Horm..

[26] Roginsky V.A., Stegmann H.B. (1994). Ascorbyl radical as natural indicator of oxidative stress: Quantitative regularities. Free. Radic. Biol. Med..

[27] Matsumura Y., Takagi M., Imanaka T. (1993). Regulation of *Escherichia coli* superoxide dismutase genes (*sodA* and *sodB*) by oxygen. Biotechnol. Lett..

[28] Fee J. (1991). Regulation of sod genes in *Escherichia coli*: Relevance to superoxide dismutase function. Mol. Microbiol..

[29] Doukyu N., Taguchi K. (2021). Involvement of catalase and superoxide dismutase in hydrophobic organic solvent tolerance of *Escherichia coli*. Amb Express.

[30] Sakamoto H., Touati D. (1984). Cloning of the iron superoxide dismutase gene (*sodB*) in *Escherichia coli* K-12. J. Bacteriol..

[31] Touati D. (1983). Cloning and mapping of the manganese superoxide dismutase gene (*sodA*) of *Escherichia coli* K-12. J. Bacteriol..

[32] Imlay K., Imlay J.A. (1996). Cloning and analysis of sodC, encoding the copper-zinc superoxide dismutase of *Escherichia coli*. J. Bacteriol..

[33] Hopkin K.A., Papazian M.A., Steinman H.M. (1992). Functional differences between manganese and iron superoxide dismutases in *Escherichia coli* K-12. J. Biol. Chem..

[34] Schellhorn H. (1995). Regulation of hydroperoxidase (catalase) expression in *Escherichia coli*. FEMS Microbiol. Lett..

[35] von Ossowski I., Mulvey M.R., Leco P.A., Borys A., Loewen P.C. (1991). Nucleotide sequence of *Escherichia coli katE*, which encodes catalase HPII. J. Bacteriol..

[36] Brown S.M., Howell M.L., Vasil M.L., Anderson A.J., Hassett D.J. (1995). Cloning and characterization of the *katB* gene of *Pseudomonas aeruginosa* encoding a hydrogen peroxide-inducible catalase: Purification of KatB, cellular localization, and demonstration that it is essential for optimal resistance to hydrogen peroxide. J. Bacteriol..

[37] Hillar A., Van Caeseele L., Loewen P.C. (1999). Intracellular location of catalase-peroxidase hydroperoxidase I of *Escherichia coli*. FEMS Microbiol. Lett..

[38] Heimberger A., Eisenstark A. (1988). Compartmentalization of catalases in *Escherichia coli*. Biochem. Biophys. Res. Commun..

[39] Imlay J., Fridovich I. (1991). Assay of metabolic superoxide production in *Escherichia coli*. J. Biol. Chem..

[40] Imlay J.A. (2008). Cellular defenses against superoxide and hydrogen peroxide. Annu. Rev. Biochem..

[41] Miscevic D., Mao J.Y., Moo-Young M., Chou C.H.P. (2020). High-level heterologous production of propionate in engineered *Escherichia coli*. Biotechnol. Bioeng..

[42] Srirangan K., Liu X., Westbrook A., Akawi L., Pyne M.E., Moo-Young M., Chou C.P. (2014). Biochemical, genetic, and metabolic engineering strategies to enhance coproduction of 1-propanol and ethanol in engineered *Escherichia coli*. Appl. Microbiol. Biotechnol..

[43] Gibson D.G., Young L., Chuang R.-Y., Venter J.C., Hutchison C.A., Smith H.O. (2009). Enzymatic assembly of DNA molecules up to several hundred kilobases. Nat. Methods.

[44] Neidhardt F.C., Bloch P.L., Smith D.F. (1974). Culture medium for enterobacteria. J. Bacteriol..

[45] Mauzerall D., Granick S. (1956). The occurrence and determination of δ-aminolevulinic acid and porphobilinogen in urine. J. Biol. Chem..

[46] Shivaprasad D., Taneja N.K., Lakra A., Sachdev D. (2021). In vitro and in situ abrogation of biofilm formation in *E. coli* by vitamin C through ROS generation, disruption of quorum sensing and exopolysaccharide production. Food Chem..

[47] Tajkarimi M., Ibrahim S.A. (2011). Antimicrobial activity of ascorbic acid alone or in combination with lactic acid on *Escherichia coli* O157: H7 in laboratory medium and carrot juice. Food Control.

[48] Tabak M., Armon R., Rosenblat G., Stermer E., Neeman I. (2003). Diverse effects of ascorbic acid and palmitoyl ascorbate on Helicobacter pylori survival and growth. FEMS Microbiol. Lett..

[49] Arrigoni O., De Tullio M.C. (2002). Ascorbic acid: Much more than just an antioxidant. Biochim. Et Biophys. Acta (BBA)-Gen. Subj..

[50] Pu W., Chen J., Zhou Y., Qiu H., Shi T., Zhou W., Guo X., Cai N., Tan Z., Liu J. (2023). Systems metabolic engineering of *Escherichia coli* for hyper-production of 5-aminolevulinic acid. Biotechnol. Biofuels Bioprod..

[51] Couto M.R., Rodrigues J.L., Braga A., Dias O., Rodrigues L.R. (2024). Optimization of chondroitin production in *E. coli* using genome scale models. Mol. Syst. Des. Eng..

[52] Zhang S., He Y., Sen B., Chen X., Xie Y., Keasling J.D., Wang G. (2018). Alleviation of reactive oxygen species enhances PUFA accumulation in *Schizochytrium* sp. through regulating genes involved in lipid metabolism. Metab. Eng. Commun..

[53] Esteve-Gassent M.D., Smith T.C., Small C.M., Thomas D.P., Seshu J. (2015). Absence of sodA increases the levels of oxidation of key metabolic determinants of *Borrelia burgdorferi*. PLoS ONE.

[54] Gort A.S., Ferber D.M., Imlay J.A. (1999). The regulation and role of the periplasmic copper, zinc superoxide dismutase of *Escherichia coli*. Mol. Microbiol..

[55] Mohandass S., Ragavan M., Gnanasekaran D., Lakshmanan U., Dharmar P., Saha S.K. (2021). Overexpression of Cu/Zn superoxide dismutase (Cu/Zn SOD) in *Synechococcus elongatus* PCC 7942 for enhanced azo dye removal through hydrogen peroxide accumulation. Biology.

[56] Panek H.R., O’Brian M.R. (2004). KatG is the primary detoxifier of hydrogen peroxide produced by aerobic metabolism in *Bradyrhizobium japonicum*. J. Bacteriol..

[57] Beas J.Z., Videira M.A., Saraiva L.M. (2022). Regulation of bacterial haem biosynthesis. Coord. Chem. Rev..

[58] Benov L., Sequeira F. (2003). *Escherichia coli* ∆ *fur* mutant displays low HPII catalase activity in stationary phase. Redox Rep..

[59] Mishra S., Imlay J. (2012). Why do bacteria use so many enzymes to scavenge hydrogen peroxide?. Arch. Biochem. Biophys..

[60] Mancini S., Imlay J.A. (2015). The induction of two biosynthetic enzymes helps *E scherichia coli* sustain heme synthesis and activate catalase during hydrogen peroxide stress. Mol. Microbiol..

[61] Geng Z., Ge J., Cui W., Zhou H., Deng J., Xu B. (2022). Efficient de novo biosynthesis of heme by membrane engineering in *Escherichia coli*. Int. J. Mol. Sci..

[62] Yang S., Guo Z., Sun J., Wei J., Ma Q., Gao X. (2024). Recent advances in microbial synthesis of free heme. Appl. Microbiol. Biotechnol..

[63] Chen H., Wang Y., Wang W., Cao T., Zhang L., Wang Z., Chi X., Shi T., Wang H., He X. (2024). High-yield porphyrin production through metabolic engineering and biocatalysis. Nat. Biotechnol..

