# Supplementary material for: Manipulating Intracellular Oxidative Conditions to Enhance Porphyrin Production in Escherichia coli
Source: Bioengineering (Basel). 2025 Jan 17;12(1):83. doi: 10.3390/bioengineering12010083 (PMC11763182; doi:10.3390/bioengineering12010083)
Supplement: Supplementary file 1 [file bioengineering-12-00083-s001.zip › bioengineering-3412263-supplementary.pdf]

Supplementary materials

Manipulating intracellular oxidative conditions for enhanced porphyrin production in *Escherichia coli*

Bahareh Arab, Murray Moo-Young, Yilan Liu, C. Perry Chou*

Department of Chemical Engineering

University of Waterloo

Waterloo, Ontario, Canada N2L 3G1

* Corresponding author:
C. Perry Chou
Department of Chemical Engineering
University of Waterloo
200 University Avenue West
Waterloo, Ontario, Canada N2L 3G1
E-mail: cpchou@uwaterloo.ca
Telephone: +1-519-888-4567 (ext. 33310)

Table S1: Oligomers used in this study

Primers/oligo name	Primer/oligo sequence (5' → 3')
P1	AAGGAGGAATAGAAAATGGACTACAATCTGGCACTCG
P2	ATGTAAATTTCCTCCTTTGGTACCTTAACGCAGAATCTTCTTCTCAG
P3	TAATTAAGTAAGGATCCGCAGCCCGCCTAATGAG
P4	GTAGTCCATTTTCTATTCCTCCTTTAATTGTTATCCGCTC
P5	TACCAAAGGAGGAAATTTACATGAGCTATACCCTGCCATCC
P6	ATAATAATCCTCCTTTGCATGCTTATTTTTTCGCCGCAAAACGTG
P7	GCATGCAAAGGAGGATTATTATATGTCATTCGAATTACCTGC
P8	CTATTTAACCTCCTTTACTAGTTTATGCAGCGAGATTTTTCG
P9	ACTAGTAAAGGAGGTTAAATAGATGAAACGTTTTAGTCTGGC
P10	GCTGCGGATCCTTACTTAATTACACCACAGG
P11	CGAAAAAATAAGGATCCGCAGCCCGCCTAATGAG
P12	GCTGCGGATCCTTATTTTTTCGCCGCAAAACGTG
P13	TCGCTGCATAAGGATCCGCAGCCCGCCTAATGAG
P14	TACCAAAGGAGGAAATTTACATGTCATTCGAATTACCTGC
P15	GCTGCGGATCCTTATGCAGCGAGATTTTTCG
P16	TACCAAAGGAGGAAATTTACATGAAACGTTTTAGTCTGGC
P17	ACCTGCTGTAAGGATCCGCAGCCCGCCTAATGAG
P18	TACCAAAGGAGGAAATTTACATGAGC
P19	CTTTTCGGATGTTACTTAATTACACCACAGGCATAGCG
P20	TAATTAAGTAACATCCGAAAAGAATGATGGATC
P21	TGTTGCGACATTATTGATTCCTCCTTTAATTGGG
P22	AGGAATCAATAATGTCGCAACATAACGAAAAGAACC
P23	TGAATTTTCCTCCTTTTCATGATCAGGCAGGAATTTTGTCAATCTTAGG
P24	TCATGAAAAGGAGGAAAATTCAATGAGCACGTCAGACGATATCC
P25	GCTGCGGATCCTTACAGCAGGTCGAAACGGTC
P26	GGAGGAATAGAAAATGGACTACAATCTGG
P27	TTCCTGCCTGAGGATCCGCAGCCCGCCTAATGAG
P28	GCTGCGGATCCTCAGGCAGGAATTTTGTCAATCTTAGG
P29	GACGTGCTCATTATTGATTCCTCCTTTAATTGGG
P30	AGGAATCAATAATGAGCACGTCAGACGATATCC
P31	CTTTTCGGATGTTATTTTTTCGCCGCAAAACGTG
P32	CGAAAAAATAACATCCGAAAAGAATGATGGATC

Table S2: Statistical analysis data for the UP-III/UP-I ratio for 0.5 g/L ascorbic acid supplementation vs the control strain

t-Test: Paired Two Sample for Means (α = 0.05)

	Control	*0.5 g/L Ascorbic acid*
Mean	0.62764495	1.44557979
Variance	0.00868759	0.00282797
Observations	2	2
Pearson Correlation	-1	
Hypothesized Mean Difference	0	
df	1	
t Stat	-7.9019515	
P(T<=t) one-tail	**0.04006944**	
t Critical one-tail	6.31375151	
P(T<=t) two-tail	0.08013888	
t Critical two-tail	12.7062047	

Table S3: Statistical analysis data for the UP-III/UP-I ratio for 1 g/L ascorbic acid supplementation vs the control strain

t-Test: Paired Two Sample for Means (α = 0.05)

	Control	*1 g/L Ascorbic acid*
Mean	0.62764495	2.57398428
Variance	0.00868759	0.00896544
Observations	2	2
Pearson Correlation	-1	
Hypothesized Mean Difference	0	
df	1	
t Stat	-14.649488	
P(T<=t) one-tail	**0.02169474**	
t Critical one-tail	6.31375151	
P(T<=t) two-tail	0.04338948	
t Critical two-tail	12.7062047	

Table S4: Statistical analysis data for the UP-III/UP-I ratio for 2 g/L ascorbic acid supplementation vs the 0.5 g/L ascorbic acid supplementation

t-Test: Paired Two Sample for Means (α = 0.05)

	0.5 g/L Ascorbic acid	*2 g/L Ascorbic acid*
Mean	1.44557979	1.5469349
Variance	0.00282797	0.04561101
Observations	2	2
Pearson Correlation	-1	
Hypothesized Mean Difference	0	
df	1	
t Stat	-0.5373567	
P(T<=t) one-tail	**0.34304634**	
t Critical one-tail	6.31375151	
P(T<=t) two-tail	0.68609268	
t Critical two-tail	12.7062047	

Table S5: Statistical analysis data for the UP-III/UP-I ratio for SOD2 vs the control strain

t-Test: Paired Two Sample for Means (α = 0.05)

	Control	*SOD2*
Mean	0.62764495	1.94063513
Variance	0.00868759	0.01188752
Observations	2	2
Pearson Correlation	1	
Hypothesized Mean Difference	0	
df	1	
t Stat	-117.35372	
P(T<=t) one-tail	**0.00271233**	
t Critical one-tail	6.31375151	
P(T<=t) two-tail	0.00542466	
t Critical two-tail	12.7062047	

Table S6: Statistical analysis data for the UP-III/UP-I ratio for SOD3 vs the control strain

t-Test: Paired Two Sample for Means (α = 0.05)

	Control	*SOD3*
Mean	0.62764495	1.75227929
Variance	0.00868759	9.6012E-05
Observations	2	2
Pearson Correlation	-1	
Hypothesized Mean Difference	0	
df	1	
t Stat	-15.440617	
P(T<=t) one-tail	**0.02058635**	
t Critical one-tail	6.31375151	
P(T<=t) two-tail	0.0411727	
t Critical two-tail	12.7062047	

Table S7: Statistical analysis data for the UP-III/UP-I ratio for SOD2 + 1 g/L ascorbic acid supplementation vs the control strain

t-Test: Paired Two Sample for Means (α = 0.05)

	Control	*SOD2 + 1 g/L Ascorbic acid*
Mean	0.62764495	1.4131059
Variance	0.00868759	0.00014286
Observations	2	2
Pearson Correlation	-1	
Hypothesized Mean Difference	0	
df	1	
t Stat	-10.563096	
P(T<=t) one-tail	**0.0300446**	
t Critical one-tail	6.31375151	
P(T<=t) two-tail	0.06008921	
t Critical two-tail	12.7062047	

Table S8: Statistical analysis data for the UP-III/UP-I ratio for KAT1 vs the control strain

t-Test: Paired Two Sample for Means (α = 0.05)

	Control	*KAT1*
Mean	0.62764495	6.20477723
Variance	0.00868759	0.03422162
Observations	2	2
Pearson Correlation	1	
Hypothesized Mean Difference	0	
df	1	
t Stat	-85.933143	
P(T<=t) one-tail	**0.00370399**	
t Critical one-tail	6.31375151	
P(T<=t) two-tail	0.00740798	
t Critical two-tail	12.7062047	

Table S9: Statistical analysis data for the UP-III/UP-I ratio for KAT2 vs the control strain

t-Test: Paired Two Sample for Means (α = 0.05)

	Control	*KAT2*
Mean	0.62764495	1.28336217
Variance	0.00868759	0.00098766
Observations	2	2
Pearson Correlation	-1	
Hypothesized Mean Difference	0	
df	1	
t Stat	-7.4403655	
P(T<=t) one-tail	**0.04252664**	
t Critical one-tail	6.31375151	
P(T<=t) two-tail	0.08505328	
t Critical two-tail	12.7062047	

List of Abbreviations

5-ALA	5 Aminolevulinic Acid
CPG-I	Coproporphyrinogen I
CPG-III	Coproporphyrinogen III
CP-I	Coproporphyrin I
CP-III	Coproporphyrin III
HemA	5 Aminolevulinate Synthase
HemB	Porphobilinogen Synthase
HemC	Porphobilinogen Deaminase
HemD	Uroporphyrinogen III Synthase
HemE	Uroporphyrinogen Decarboxylase
HemF	Coproporphyrinogen III Oxidase
HemG	Protoporphyrinogen Oxidase
HemH	Protoporphyrin Ferrochelatase
HemN	Oxygen Independent Coproporphyrinogen III Oxidase
HMB	Hydroxymethylbilane
IPTG	Isopropyl β-D-1-Thiogalactopyranoside
KatE	Catalase HPII
KatG	Catalase Peroxidase HPI
OD600	Optical Density at 600 nm
PBG	Porphobilinogen
PP-IX	Protoporphyrin IX
PPG-IX	Protoporphyrinogen IX
ROS	Reactive Oxygen Species
RBS	Ribosome Binding Site
SOD	Superoxide Dismutase
UPG-I	Uroporphyrinogen I
UPG-III	Uroporphyrinogen III

UP-I	Uroporphyrin I
UP-III	Uroporphyrin III
